# Development of a Consensus Taxonomy of Sedentary Behaviors (SIT): Report of Delphi Round 1

**DOI:** 10.1371/journal.pone.0082313

**Published:** 2013-12-02

**Authors:** Sebastien Francois Martin Chastin, Ulf Schwarz, Dawn Ann Skelton

**Affiliations:** 1 School of Health and life Science, Institute of Applied Health Research, Glasgow Caledonian University, Glasgow, United Kingdom; 2 Institute of Formal Ontology and Medical Information Science, Saarland University, Saarbrücken, Germany; UCSD School of Medicine, United States of America

## Abstract

**Background:**

Over the last decade, sedentary behaviors have emerged as a distinctive behavioral paradigm with deleterious effects on health independent of physical activity. The next phase of research is to establish dose response between sedentary behaviors and health outcomes and improve understanding of context and determinants of these behaviors. Establishing a common taxonomy of these behaviors is a necessary step in this process.

**Aim:**

The Sedentary behavior International Taxonomy project was developed to establish a classification of sedentary behaviors by use of a formal consensus process.

**Methods:**

The study follows a Delphi process in three Rounds. A preparatory stage informed the development of terms of reference documents. In Round 1, experts were asked to make statements about the taxonomy; 1) its purpose and use ; 2) the domains, categories or facets that should be consider and include; 3) the structure/architecture to arrange and link these domains and facets. In Round 2 experts will be presented with a draft taxonomy emerging from Round 1 and invited to comment and propose alterations. The taxonomy will then be finalised at the outset of this stage.

**Results:**

Results of Round 1 are reported here. There is a general consensus that a taxonomy will help advances in research by facilitating systematic and standardised: 1) investigation and analysis; 2) reporting and communication; 3) data pooling, comparison and meta-analysis; 4) development of measurement tools; 4) data descriptions, leading to higher quality in data querying and facilitate discoveries. There is also a consensus that such a taxonomy should be flexible to accommodate diverse purposes of use, and future advances in the field and yet provide a cross-disciplinary common language. A consensual taxonomy structure emerged with nine primary facets (Purpose, Environment, Posture, Social, Measurement, Associated behavior, Status, Time, Type) and the draft structure presented here for Round 2.

## Introduction

### Background

Sedentary behavior was first conceptualised as a lack of physical activity and the result of an inactive lifestyle [1]. More recently, it has been recognised that it is not just a lack of physical activity but an entirely separate construct, as individuals who engage in regular physical activity might also spend long periods of time sedentary at work, during transportation or leisure activities. A new and exciting field of research has emerged, treating sedentary behaviors and inactivity as related but separate paradigms. There is now mounting epidemiological, prospective and laboratory evidence that sedentary behaviors have specific and different effects on metabolism, physiology, health and well being [2] from that of a low engagement in physical activity. At the microscopic level, specific biological processes might be occurring while sedentary [3] and long continuous periods of sedentary behavior might also alter physiology [4]. At the macroscopic level, sedentary behavior appears to be associated with poor health and wellbeing outcomes [5] and is a risk factor for all-cause mortality [6], independent of levels of physical activity. Interventions to increase physical activity do not systematically impact the time spent sedentary [7]. Consequently, policies concerning sedentary behavior have started to appear. The WHO and several countries (e.g. Canada, UK, Ireland, New Zealand, France and USA) have issued recommendations to limit sedentary time at all ages [8].

### Future of sedentary behavior research and the need for a taxonomy

In order to refine these guidelines and strengthen the evidence base, further research is required. In particular, causal and dose-response relationships between sedentary behaviors and health need to be established. This relies on a better understanding of determinants and context of these behaviors, to develop good quality intervention trials [9]. In turn, this can only be achieved effectively if a clear definition, common terminology and taxonomy standardising and harmonising data, measurements and outcomes are integral part of the research process [10]. This process is one which has enabled major advances in the field of falls prevention and has allowed pooling and meta-analysis of data, standardised measurement and reporting of outcome measures and interventions [11][12][13].

A common operational definition of sedentary behavior has slowly emerged from one purely based on an energy-expenditure paradigm [14] to one taking into account posture, as more authors now talk about sedentary behavior being equal to sitting. A number of policy documents have adopted the definition (or very similar), “*Sedentary behaviors are behaviors characterised by a seated or reclining posture and low energy expenditure ≤ 1.5 MET during waking hours*” [15], and there has been a recent call, signed by 52 researchers, to use this standardised terminology in the literature [16]. 

This definition clearly sets sedentary behavior as a distinct paradigm from the lack of physical activity and should prevent further confusion between sedentary behavior and inactivity. This is an important first step but the definition alone is not sufficient to enable the required advances in research. The development of a taxonomy is required in addition to this definition for three main reasons; to eliminate confusion over terminology and meaning, to provide a structure for the current and future knowledge of sedentary behavior and to provide a basis to distinguish different behaviors. 

The current definition has a dual ontology - energy consumption on one hand and posture on the other - which still leaves room for confusion. For example it is not clear whether a brief sleep during the day should be considered a sedentary behavior or not, while lying down and listening to music without moving, clearly should be. The definition also describes a very broad range of behaviors indiscriminately. However, recent evidence suggests that different types of sedentary behaviors have different impacts on health and wellbeing [17] [18]. It is also conceivable that some sedentary behaviors might have health-enhancing effects (we all need to rest and relax sometimes and this might have salutogenic effects). Therefore, some authors [17] [18] argue for a more precise classification and monitoring system in order to identify the effect of different behaviors. Similarly, different types of sedentary behavior might be more or less difficult to modify in different contexts [1]. Distinguishing which behavior to target and develop effective interventions to modify it cannot be realised using the above definition alone but requires more precise information. In addition, a number of international projects, such as the European Joint Initiative on the Determinants of Diet and Physical Activity (DEDIPAC www.healthydietforhealthylife.eu/index.php/joint-actions/joint-action-1-dedipac) seek to create large databases of information and aggregate data on sedentary behavior in order to advance public health research through data re-use, secondary analysis and harmonised surveillance systems. The current definition of sedentary behavior alone is not sufficient to enable the harmonised collection, organisation and retrieval of relevant information and data on such a large scale. Therefore the development of a taxonomy is required to enable the controlled, precise, systematic and sustainable gathering and curation of data and information on sedentary behaviors. 

### Aim

The Sedentary behavior International Taxonomy project (SIT) (www.sittonomy.net) was developed to establish classification of sedentary behaviors by use of a formal consensus. This article reports the results of the initial Round of this process and invites engagement in the final developmental stage.

## Methods

### Process

**Figure 1:**
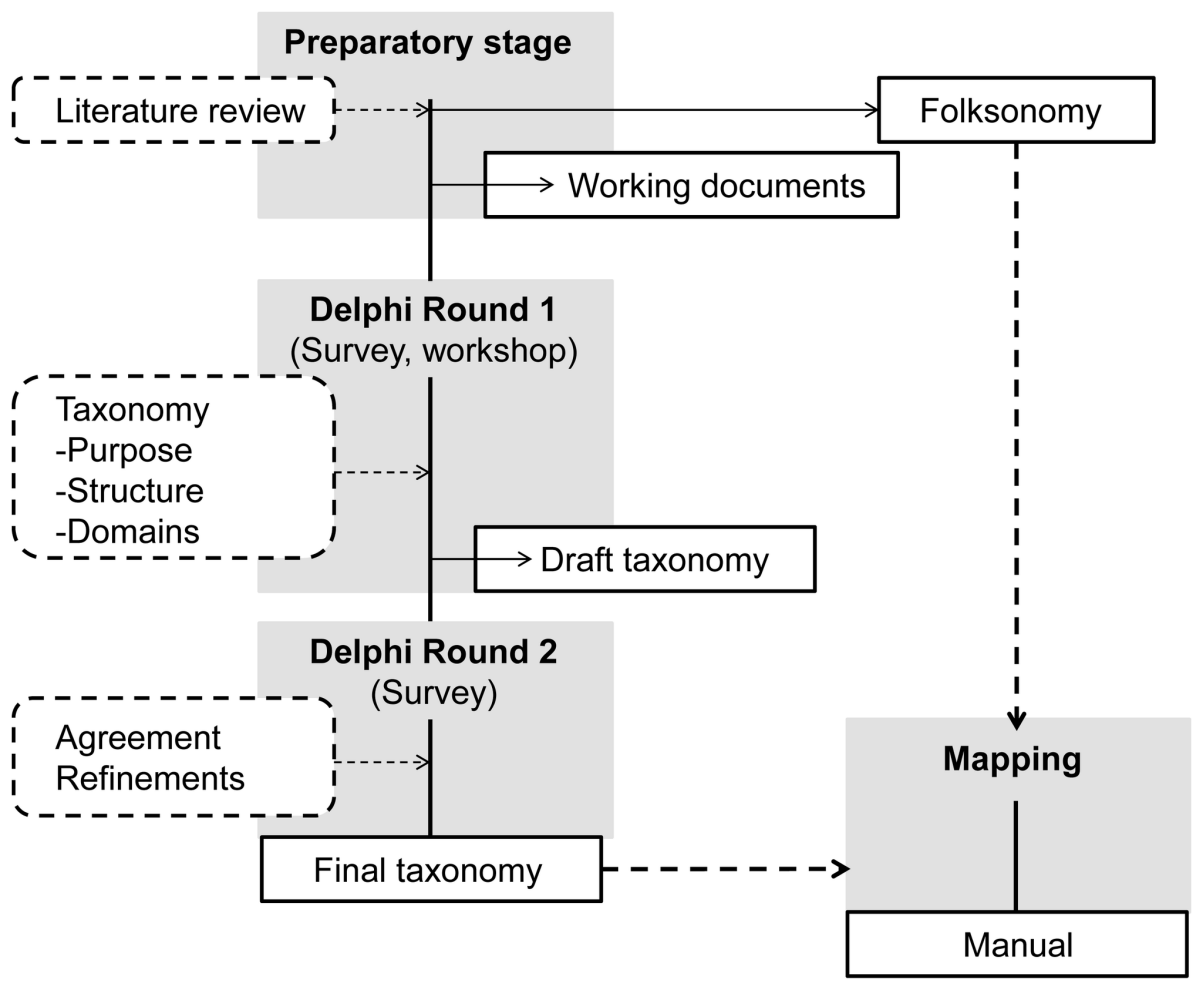
Taxonomy development flow.

The development of the taxonomy follows a Delphi expert consensus process [19][20] (Figure 1). In the first step (preparatory stage), a systematic search of the literature published on sedentary behaviors was conducted within electronic databases (December 2011: AMED; Cochrane Library; CINAHL; Medline) to guide the development of working documents and identify experts. 

These documents detailed the following: the current definitions of sedentary behavior; the possible role and use of a taxonomy; the types of possible architecture for the taxonomy (detailed in the supporting material Text S1 and Figure S1) which required consensus; and the consensus process methodology. In addition, a simple skeletal taxonomy was included in the documents to serve as a basis for discussion. These working documents were made available to all experts who were contacted and those who registered on the project website (www.sittonomy.net). 

In Round 1 experts were asked to make statements about: 1) the purpose and possible use of the taxonomy; 2) the domains/categories or facets the taxonomy should consider and include; 3) the structure/architecture which the taxonomy should adopt to arrange and link the domains/categories and facets. In addition the experts were asked to propose a draft or model taxonomy. Responses were collected via an online survey through the website, via email and during a designated Workshop at the 8^th^ World Congress on Active Ageing [21]. Phase 1 opened in January 2012, and closed in December 2012. 

In Round 2 experts will be presented with a draft taxonomy emerging from consensus in Round 1 and invited to comment and propose alterations. The taxonomy will be finalised at the completion of this stage and a manual produced. 

A classification of sedentary behavior established from general public opinion (folksonomy) [22] runs in parallel with this expert consensus. The folksonomy is developed through tagging (labelling) of random sets of images of sedentary behavior periods by the general public [23]. Through this process a consensus around stable and shared vocabularies does emerge, even in the absence of a centrally controlled vocabulary. To date 15000 tags for 10000 images have been collected. In Round 3, the expert taxonomy will be mapped onto this folksonomy. 

### Ethics

The study was approved by Glasgow Caledonian University School of Health and Life Science Ethics Committee. All participants were given information about the study and gave written consent by completing the questionnaire. Experts were also given the opportunity to be named as contributors to the project or remain anonymous. 

### Experts

An initial list of experts was established from the list of authors of the peer-reviewed publications identified during a systematic literature search conducted in the preparatory stage as described above. These were contacted directly. Invitations were also sent to members of the Sedentary Behavior Research Network (*www.sedentarybehavior.org*), expert panel for the UK Department of Health report on sedentary behavior [15], and the UK physical activity expert consensus panel [24]. Individuals contacted were encouraged to extend the invitation to others whose expertise they deemed appropriate. Individual wishing to take part were asked to register on the project website (www.sittonomy.net) and invited to take part in the Workshop [21].

### Round 1 statement analysis

Statements were initially checked to ascertain that they fitted the definitions and method described in the working documents. Thematic analysis of the statements expressed in Round 1 was carried out. Frequency of theme appearance was used to gauge consensus and isolate areas of contention. 

Proposed categories/facets and architectures linking and arranging them into a structure were mapped to identify consensual overlap and areas of disagreement. Associations between categories/facets were studied and classed according to their relationships. Classes of relationships included; hierarchical dependency (domain/sub-domain), degree of separation (category or facets with some overlap) and mutual exclusion (domain reflecting a dichotomy). 

### Synthesis

Domains and facets were clustered according to consensus on overlap and relationships. They were then arranged according to the agreed type of structure. Frequency of appearance of the domains and facets in expert’s statements, and their relationship, guided whether they were included in the draft taxonomy. The taxonomy was then checked to ascertain that it gave an exhaustive representation of all domains and facets suggested, as well as fitting the purpose and structure highlighted by the experts involved. 

## Results

### Experts

In the preparatory stage a total of 199 experts were identified. One hundred and fifty three of the experts contacted registered to take part on the SIT website but only 69 actually expressed their opinion in Round 1. Out of these, 42 expressed their opinion via the online survey, 25 expressed their opinion at the World Congress of Active Ageing in Glasgow in August 2012 and 2 opinions were also received directly via email. These 69 individuals’ stated fields of expertise included; sedentary behavior, public health, rehabilitation science, behavioral science, gerontology, movement science, epidemiology, ergonomics, medicine, occupational health, measurement and instrumentation, sociology of medicine, sociology of public health, sociology of ageing, ageing, mobility, cardiometabolic health (in children, adults and older adults), statistics, imaging, dementia, obesity, health psychology, mental health, genetics nutrition, medical imaging, physical activity intervention, physiotherapy, obesity, population health, exercise and sports, occupational health, community medicine, use of time, nutrition, genetics and biomedical engineering. 

### Purpose

There was a wide consensus (79% of respondents) that a taxonomy of sedentary behaviors will enable progress in research. A single respondent questioned the purpose of the taxonomy. The points raised were:

• Would a taxonomy be sensitive enough to the complexity of behavior capturing both the lived experience and the different contexts generating different modalities of behaviour?
• Would it shift the focus from sedentariness as a feature of individuals towards the contexts (e.g. institutional, cultural, social, environmental) in which being 'sedentary' takes place?
• Whose truth would this classification reflect?

Other respondents did not express their opinions in this matter. 

The purpose of a taxonomy of sedentary behavior put forward fell into three categories: standardisation, specificity and measurement. 

#### Standardisation

This theme was reflected in 69% of the positive statements. Experts expressed the view that a taxonomy would enable standardisation of definition, frameworks and consistency of reporting. Many experts reported already using their own form of taxonomy but would welcome harmonisation. It would facilitate comparison between studies in different populations and across the lifespan. It would also enable aggregation of results, data mining of pooled data sets resulting in more efficient research output, more reliable and stronger evidence. Finally, there was a broad consensus that it would improve communication amongst researchers and support effective data and information sharing. However some respondents pointed out that these benefits will occur only if the taxonomy is widely adopted. 

#### Specificity

This theme was present in 69% of the positive statements, reflecting consensus that being able to systematically describe sedentary behavior is needed to reliably study and further understand sedentary behaviors and their determinants. A taxonomy would allow for more specific analysis of data and assessment of health risks or benefit of specific behaviors. In turn this would result in more specific health recommendations. It would assist in understanding patterns and in identifying factors contributing to sedentary behavior, thus informing interventions. It would help in targeting specific behaviors and highlight gaps in research and identify unmet intervention needs. No statements contradicted these views.

#### Measurement

This theme was present in 35% of the positive statements. Experts expressed the view that a taxonomy could guide the development of standardised assessment tools. Clear descriptions of sedentary behavior could provide vital information for the development of new self-report tools and objective devices. No statements contradicted these views.

### Structure

Thirty two % of respondents were in favour of a faceted taxonomy, 17% leant toward a networked structure, 15% were in favour of a hierarchical structure, 15% preferred a flat structure inspired by the compendium of physical activity, 15% advocated a hybrid structure drawing elements from the different architectures and 6% preferred a more open broad framework similar to the social ecological models [9] or the International Classification of Function (ICF). In addition to the 15% who thought the taxonomy would benefit from a mixed architecture, 44% of respondents who leant toward faceted, networked and hierarchical structures commented that the taxonomy would have to draw from other architectures. Finally 75% of respondents in favour of flat architecture described or gave examples of mixed structures at subdomain level.

#### Faceted

Arguments in favour of a faceted structure were that it reflects our current state of knowledge of sedentary behaviors. A faceted structure avoids the potential danger of imposing a fixed ontology and ideas and allows refinement as the field advances. Respondents felt that it was a good structural basis to time proof the taxonomy and allow flexibility across fields of investigation and life course investigations. Facets could be linked to social ecological models or other existing classifications of behavior. The main points against a faceted structure were its potential complexity, and that it might be difficult to conceptualise and represent graphically. 

#### Networked

Respondents in favour of a networked structure made the point that it is the only structure capable of showing the association between different aspects of behavior. It is a less reductionist architecture capable of representing the complexity of behavior. It makes no assumptions about the importance of one domain above another but gives flexibility to prioritise associations between domains. Arguments expressed against a networked structure related to its conceptual complexity and potential difficulty in coding. 

#### Hierarchical

Advocates of a hierarchical structure favoured its conceptual simplicity and the fact that it is a model that is well understood and known in the scientific community. It allows the taxonomy to be used on a broad level while enabling more details to be mined if required. Epidemiologists and public health scientists proposed that a hierarchical structure can be easily linked to population surveillance structures [2]. It offers ease of representation and coding. However it was recognised that a hierarchical structure requires a deeper understanding of each domain than currently available knowledge allows. This hierarchy of domains depends on ontology and the field of investigation. There is potential danger of imposing a fixed idea derived predominantly from measurement concepts that do not capture the complexity of behavior.

#### Flat

Flat structure was advocated on the ground of its simplicity, familiarity and similarity to existing classifications of physical activity. Advocates felts that there is no need to reinvent the wheel and that a sedentary behavior classification should mirror what has been done in the field of physical activity. Criticism of the flat structure were similar to that of the hierarchical structure; reductionism, imposed and inflexible set ontology and requirement to make assumptions about domains that are not necessarily supported by the current state of knowledge. The benefit of a parallel structure with physical activity classification was debated. While this is a very pragmatic concept, some respondents voiced opinion that a flat structure potentially would influence and shape direction of investigations in the future. They invited reflection about how much the field of physical activity research has been influenced by definition of a compendium based purely on the energetic framework neglecting other social, behavioral and environmental dimensions.

#### Hybrid

There is a consensus that a hybrid structure would give more flexibility and enable a common classification across different ontologies. Positive points put forward in favour of a hybrid structure included the ability to cover broad and specific purposes. A hybrid structure based on faceted macro-level descriptors and hierarchical substructures would enable research to pick the facet of interest for a flat structure and/or aggregate them into a more hierarchical structure or to align the taxonomy to ecological models. This would allow expansion and simple structural changes with advances in the field. The consensus is that a faceted structure with hierarchical subdomains and ability to create linkages would be the most appropriate, time proof and flexible structure. Caveats highlighted are the potential complexity and difficulty in coding. 

### Categories/Facets/Domains

Statements about categories/facets/domains to describe sedentary behavior fell into three categories:

1 Categories/facets/domains describing a specific type and period of sedentary behavior. Present in 70% of experts statements;
2 Categories/facets/domains describing the personal attribute of an individual considered sedentary (e.g. their physical and mental health states). Present in 28% of expert statements;
3 Categories/facets/domains enabling to report measurements of time spent in sedentary behavior (e.g. how long, frequency [2]). Present in 19% of expert statements.

Analysis of the statement reveals nine complementary facets characterising; the purpose (why), the environment (where), the social context (with whom), the type or modality (what), associated behaviors (what else), when the behavior take place (when), the mental and functional states of sedentary individual (state), posture, measurement and quantification issues. Table 1 shows the frequency of these facets, and any overlap with other Categories/facets/domains and sub-categories. No statements contained any objection to any of these themes.

**Table 1:** Top level categories, with frequency by percentage of experts, sub-domains and link to other domains. Commas denote complementary sub-domains and / mutually exclusive sub-domains.

Facet	Frequency	Tier 1 Sub-categories	Overlap/link
Purpose	88%	Work/ education/care/ transport/ eating/ rest/relaxation/ leisure	Environment, time, social (e.g. work, leisure)
Environment	80%	Location (indoor/outdoor, built environment), physical variables (visibility, temperature), social variables	Purpose, time.
Type	58%	Screen based/not screen based	Posture
Posture	58%	Sitting, lying	Posture, measurement
Social	50%	Alone/with others (friends,family, strangers)	Environment, purpose
Time	46%	Time of day, time of year	Environment, purpose
State	38%	Functional status (limitations/none), psychological state (depression, self efficacy, emotion)	Purpose, International Classification of Function
Associated behaviors	31%	Snacking, smoking, drinking	
Measure	27%	Measurement tool (objective, self report)	SITT, time of use classifications

**Purpose** was the most frequent facet present in 88% of statements. Experts agree that it is important to describe the reason for adopting a sedentary behavior such as work, leisure or resting and whether this was volitional or enforced such as work or due to disability. Statements describing the **environment** (settings) in which a sedentary behavior takes place form the second most frequent category. These covered the location (e.g. indoors or outdoors), the type of community (e.g. urban, rural) but also the physical environment (e.g. visibility, temperature) as the first tier. These sub-categories are complementary and not mutually exclusive. More specific sub-categories including home, office, school, communal facilities, care institutions described the actual location in a dichotomous way. Specifying the **type** of sedentary behavior was the third most frequent facet. Experts presented an exhaustive list of specific activities not all mutually exclusive, however these can be classed as either screen based, or not, as first tier domains. The **posture** facet characterises whether a behavior is in a seated or reclining body position. It was frequently included as a type of sedentary behavior but the majority of experts saw it as a main facet because of its connection to measurement and association with physiological and health outcomes. Fifty % of experts proposed that the taxonomy should include a facet describing the **social** context (e.g. alone, with other including family, friends, strangers). Fourty six % of experts expressed the opinion that the timing of the behavior (**when**) should be included in the taxonomy. Proposed sub-categories included the time of the day and the time of the year. Experts also expressed the need to capture **associated behaviors** such as snacking, smoking, drinking. Experts also felt that the functional **state** (eg. frailty) of an individual should be reflected in the taxonomy. This covered two sub-categories, whether a sedentary behavior is associated with physical or mental limitations (linked to the WHO ICF), and what is the psychological state concurrent to the sedentary behavior. Finally experts expressed the opinion that the **method of measurement** capturing the sedentary behavior should be reported in the taxonomy. Some experts felt that reporting metrics such as amount of time, pattern (frequency, interruptions, duration) could also be included but the majority pronounced themselves in favour of using the taxonomy in conjunction with existing and future reporting frameworks such as SITT [2], using the taxomomy to describe the behavior. 

## Discussion

### Consensus taxonomy

**Figure 2:**
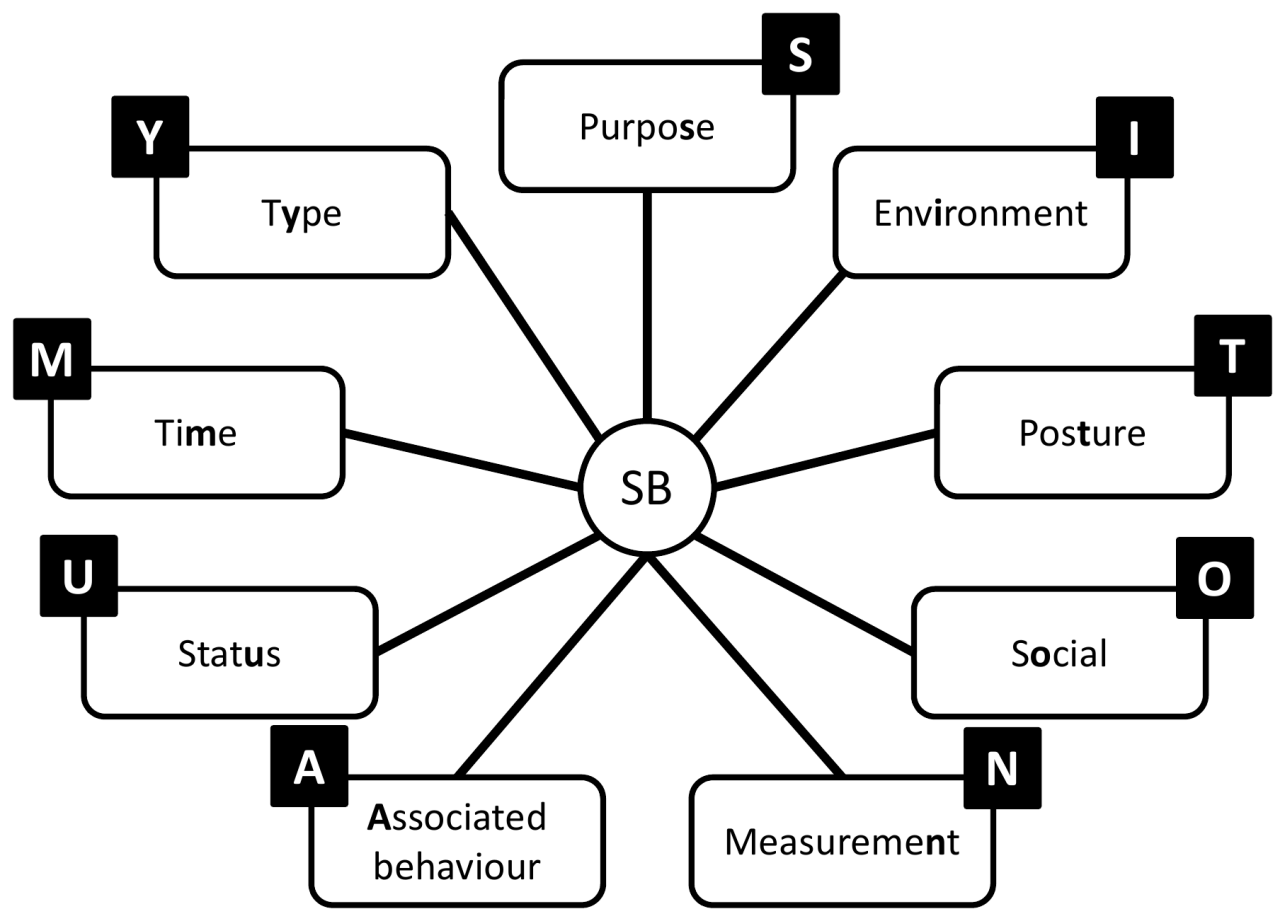
Taxonomy level one facets and coding labels.

**Figure 3:**
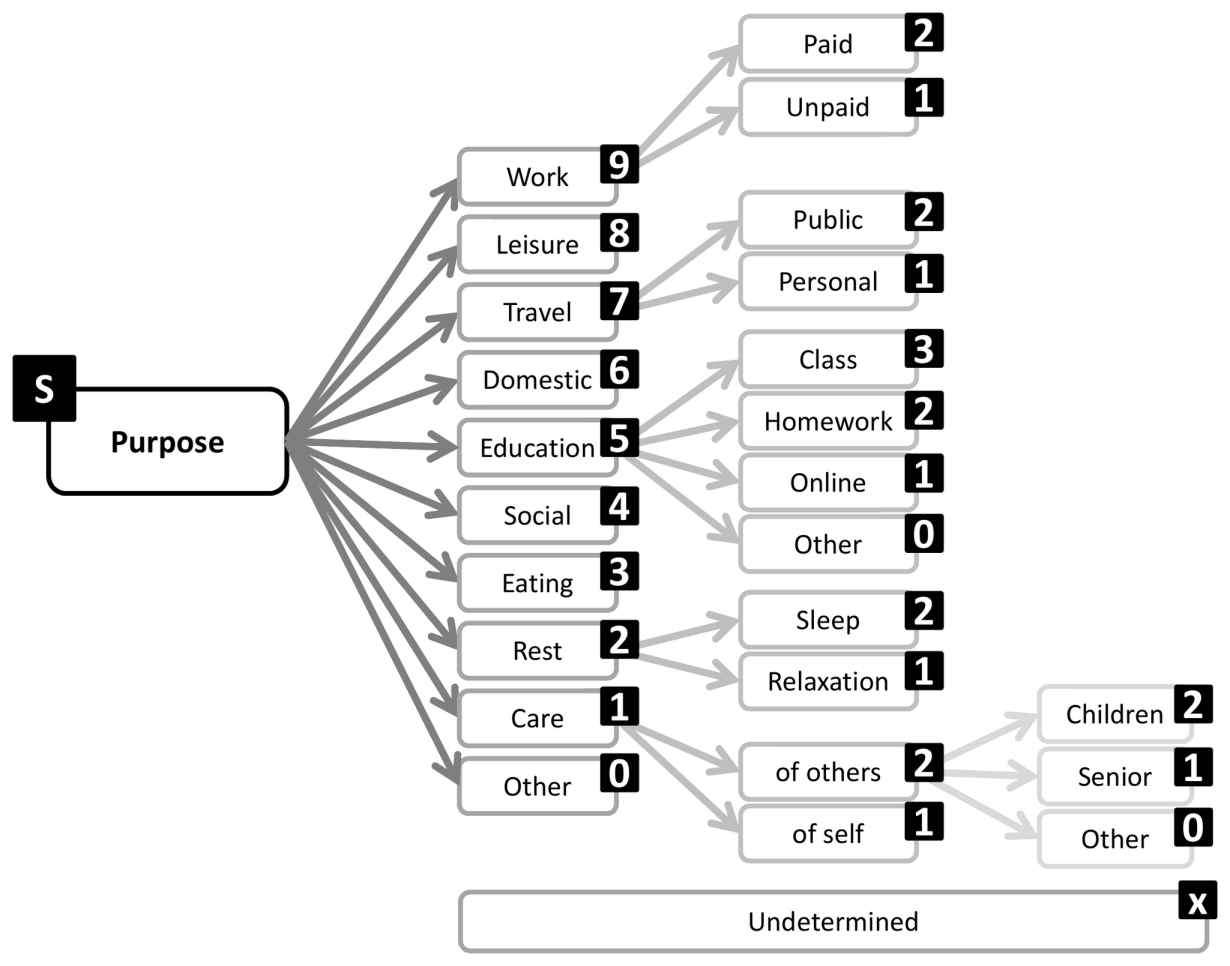
Purpose facet substructure and coding labels.

**Figure 4:**
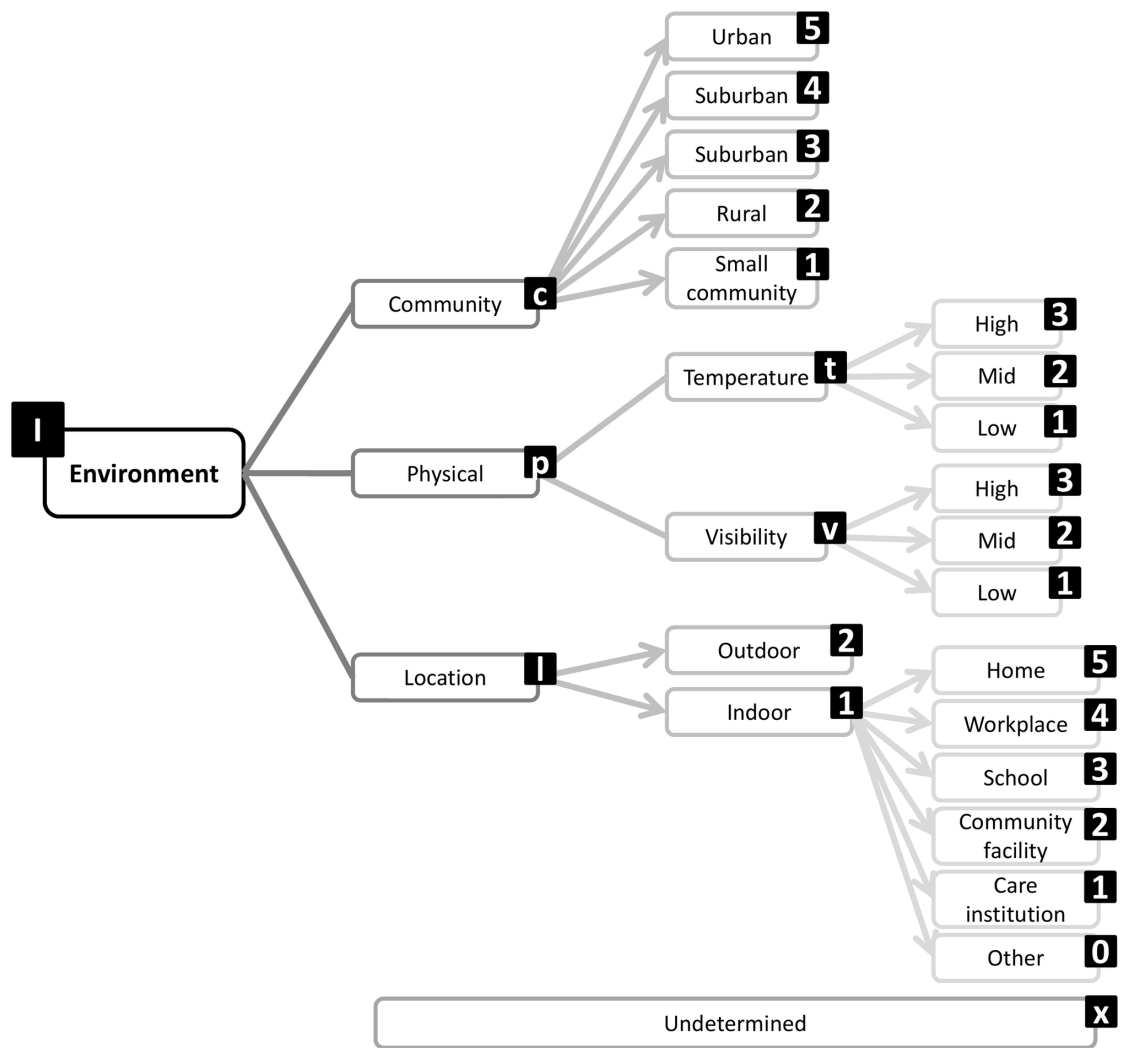
Environment facet substructure and coding labels.

**Figure 5:**
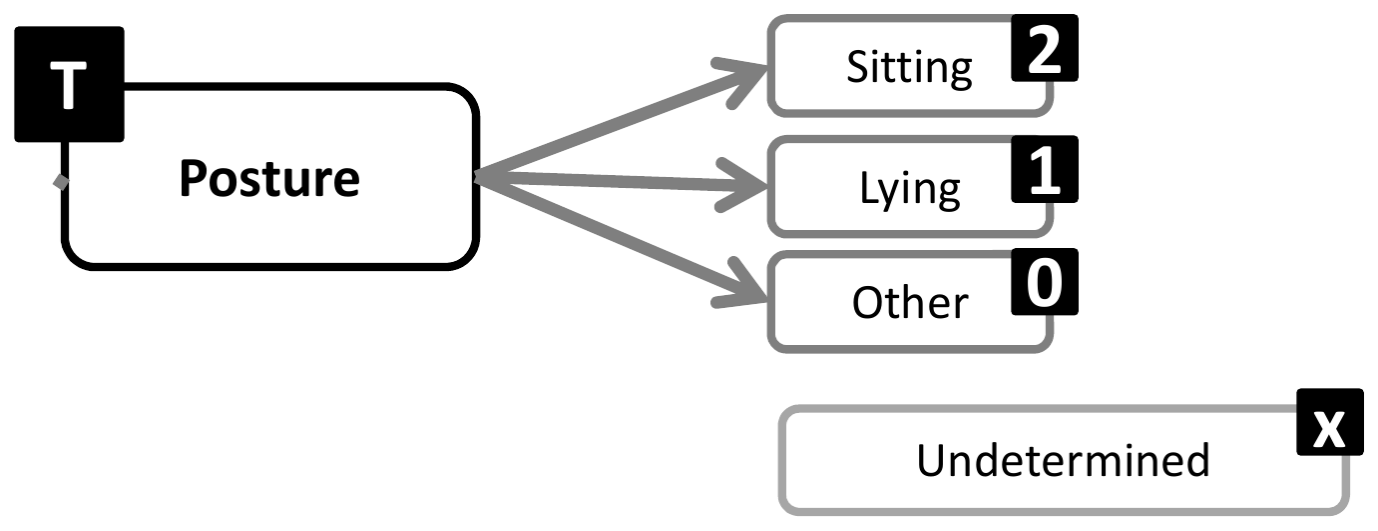
Posture facet substructure and coding labels.

**Figure 6:**
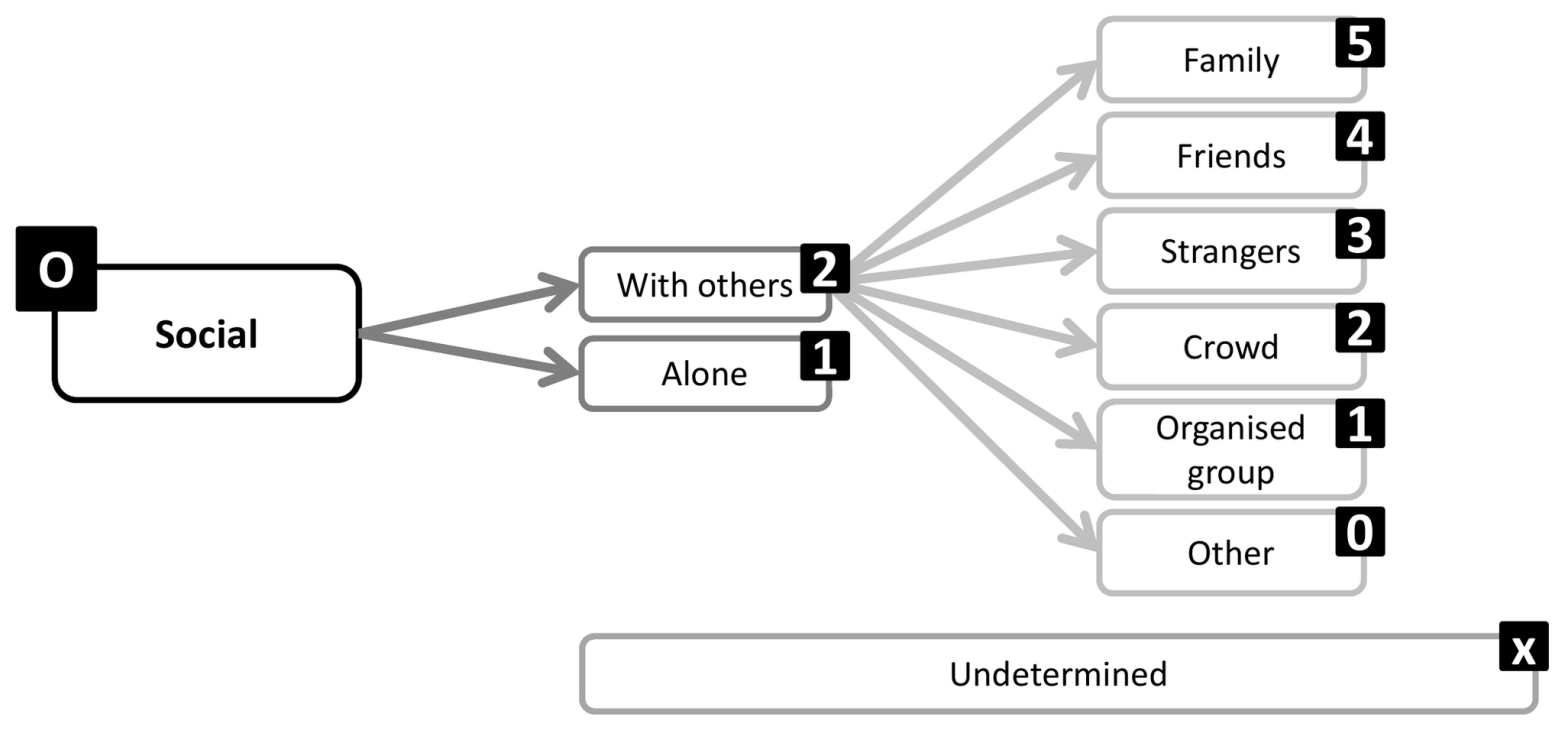
Social facet substructure and coding labels.

**Figure 7:**
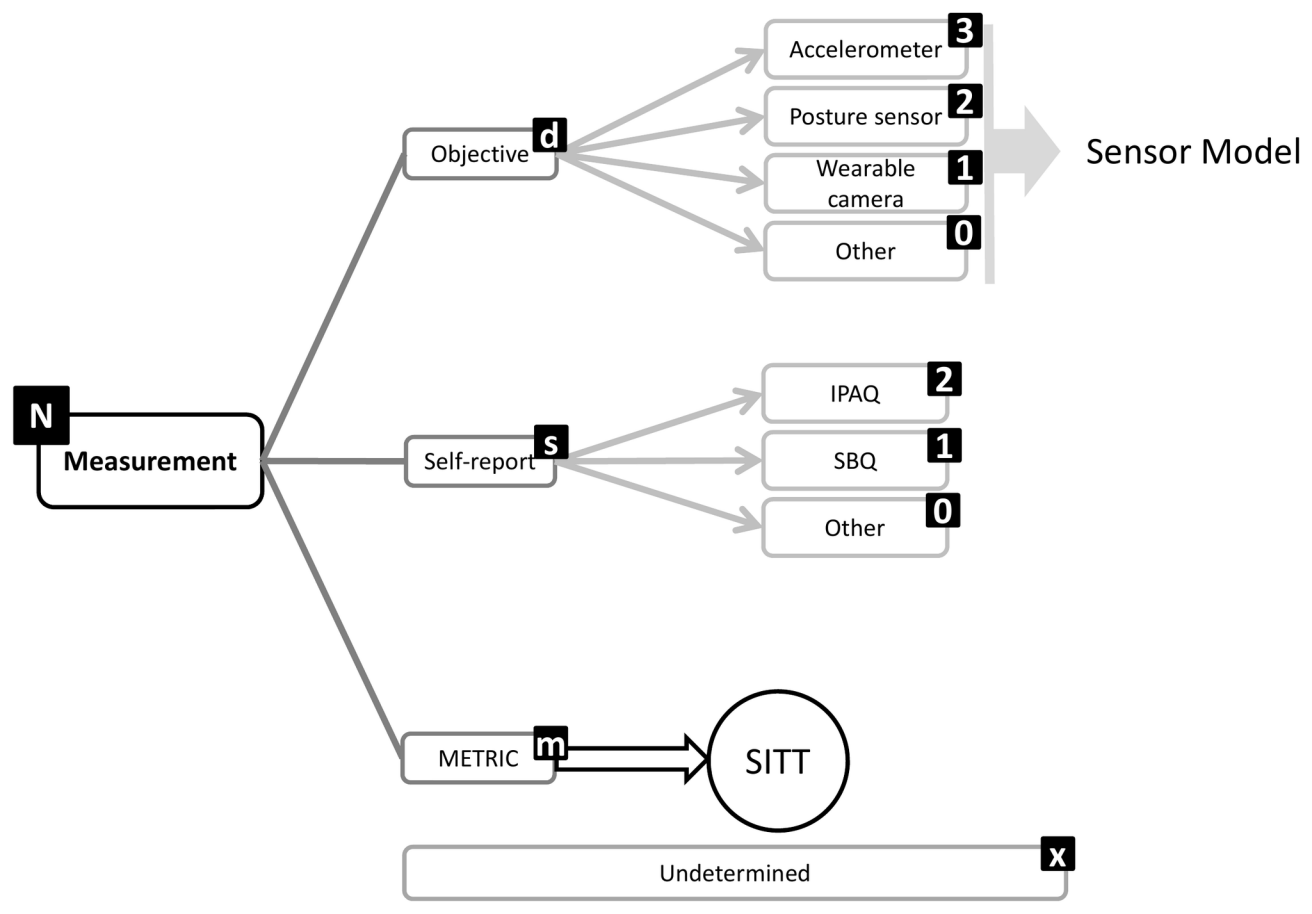
Measurement facet substructure and coding labels.

**Figure 8:**
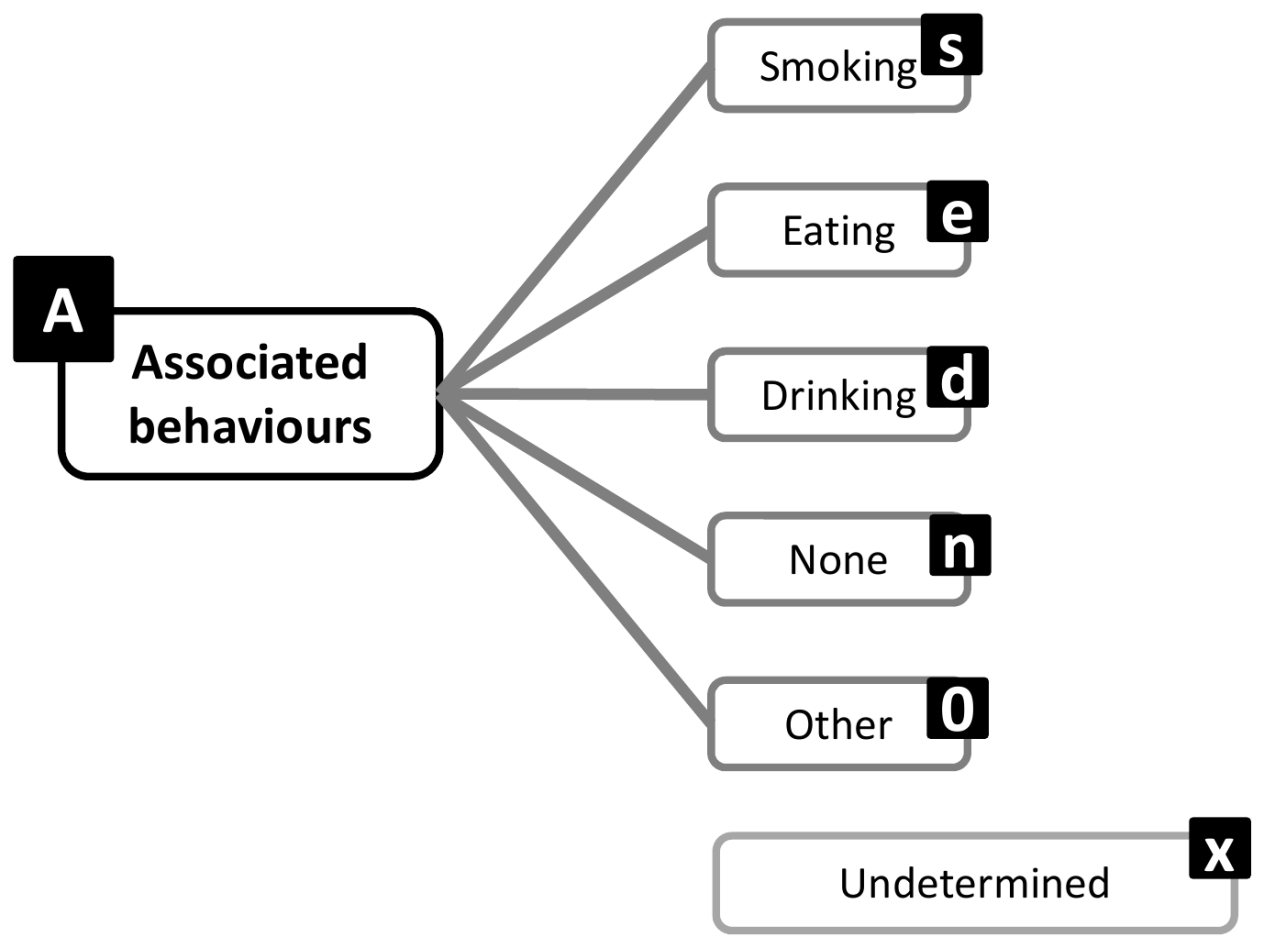
Associated behaviors facet substructure and coding labels.

**Figure 9:**
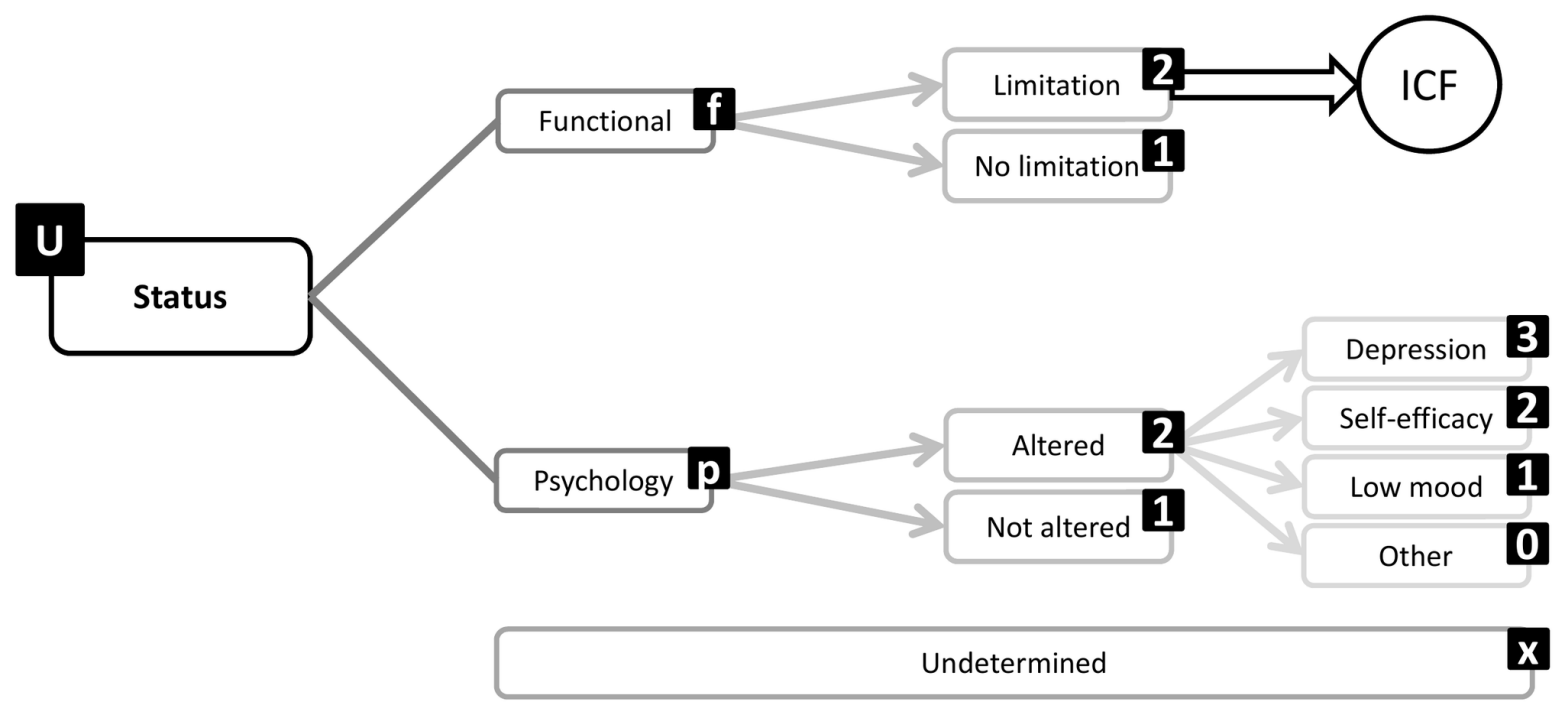
Status facet substructure and coding labels.

**Figure 10:**
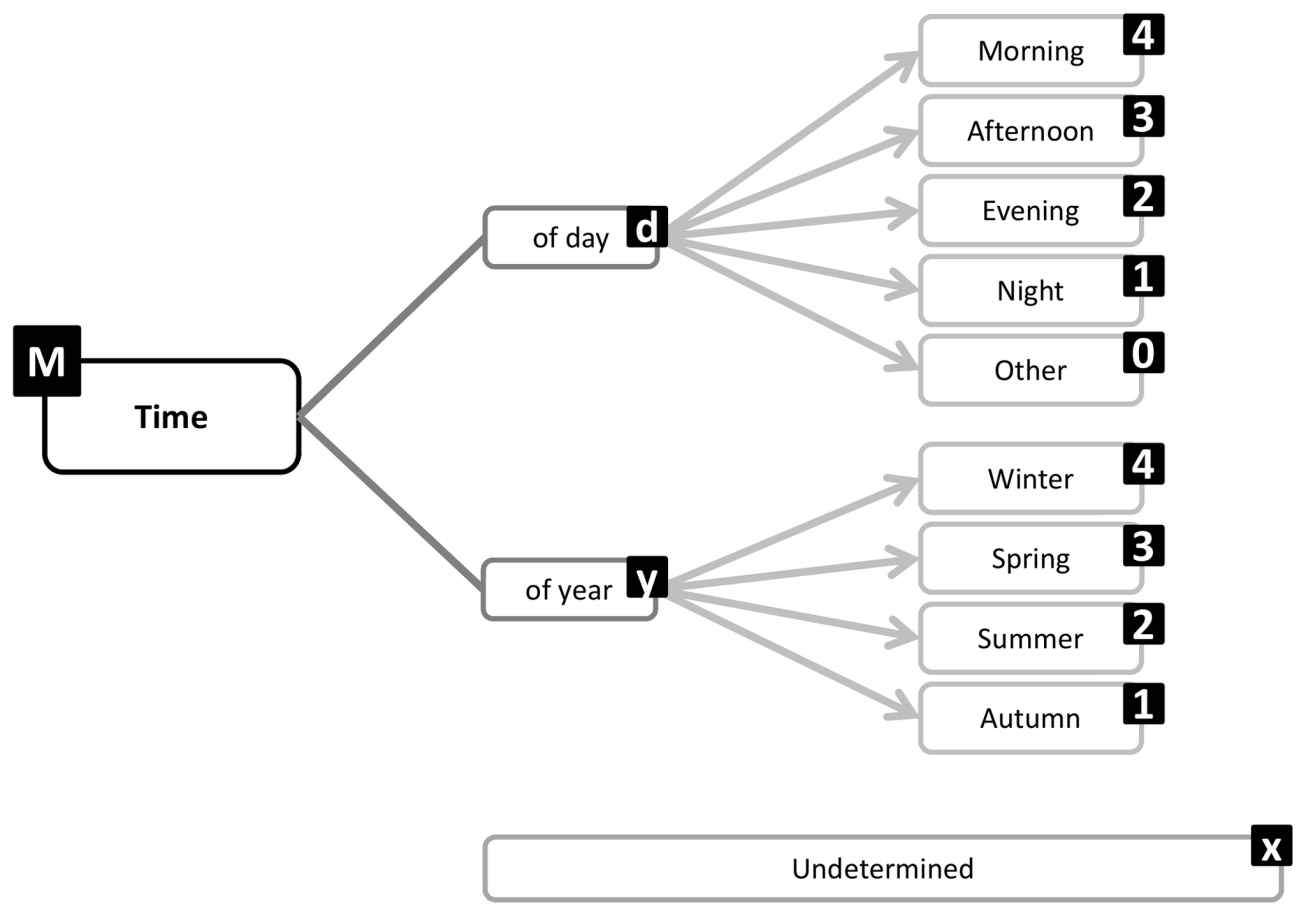
Time facet substructure and coding labels.

**Figure 11:**
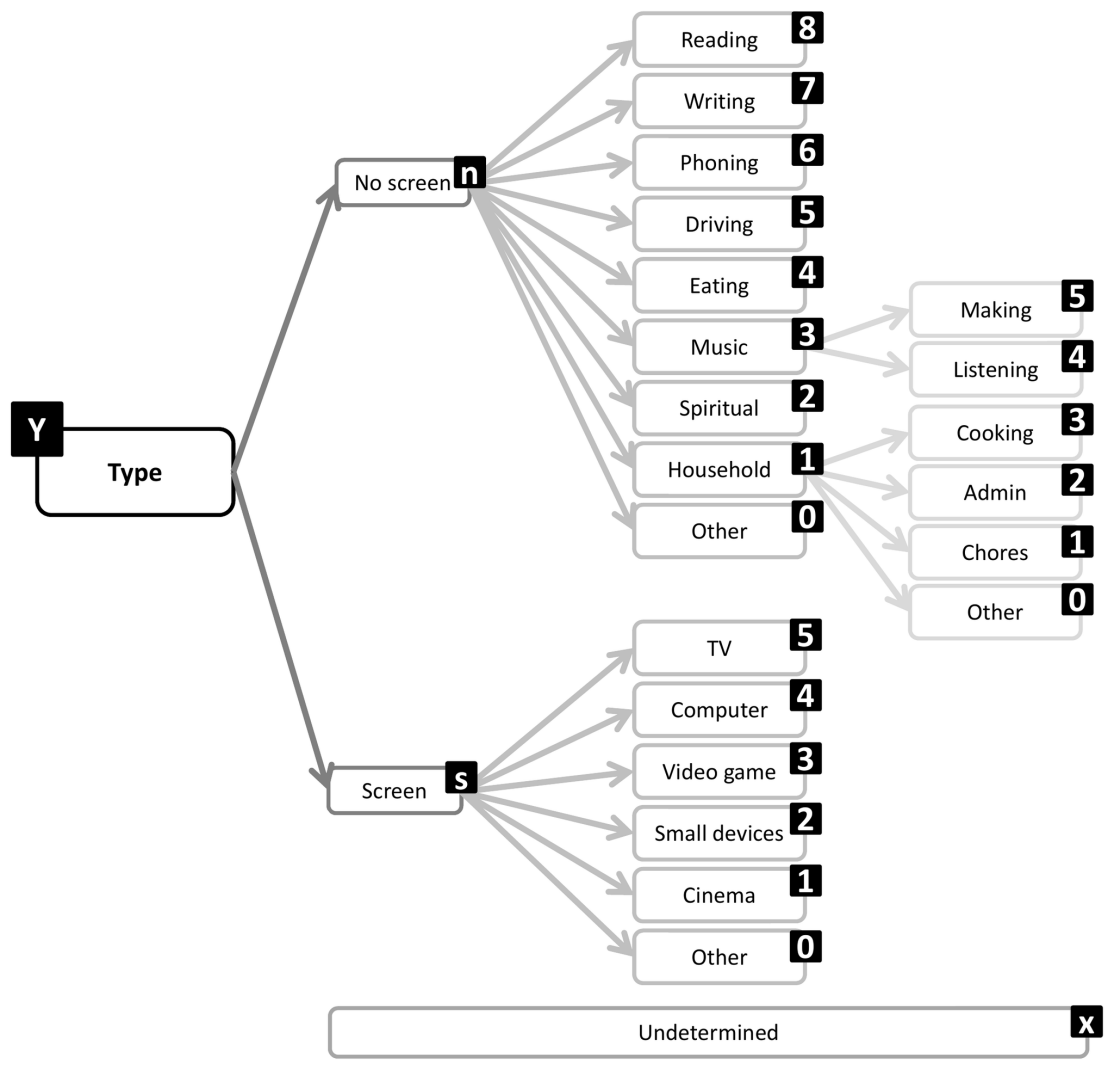
Type facet substructure and coding labels.

A structure emerged from Round 1 that enables exhaustive inclusion of all proposed categories/facets and domains. The structure is in accordance with the consensus that a hybrid architecture best suits the taxonomy. Sedentary behavior can be described by a set of nine complementary facets (Figure 2), representing the nine domains over which there is consensus. Each of these facets has sub-domains and mutually exclusive categories (Figures 3-11). The taxonomy extends over four levels and links with the WHO ICF and surveillance frameworks such as Sedentary frequency, Interruption, Time and Type (SITT)[2]. 

### Classification and coding

**Figure 12:**
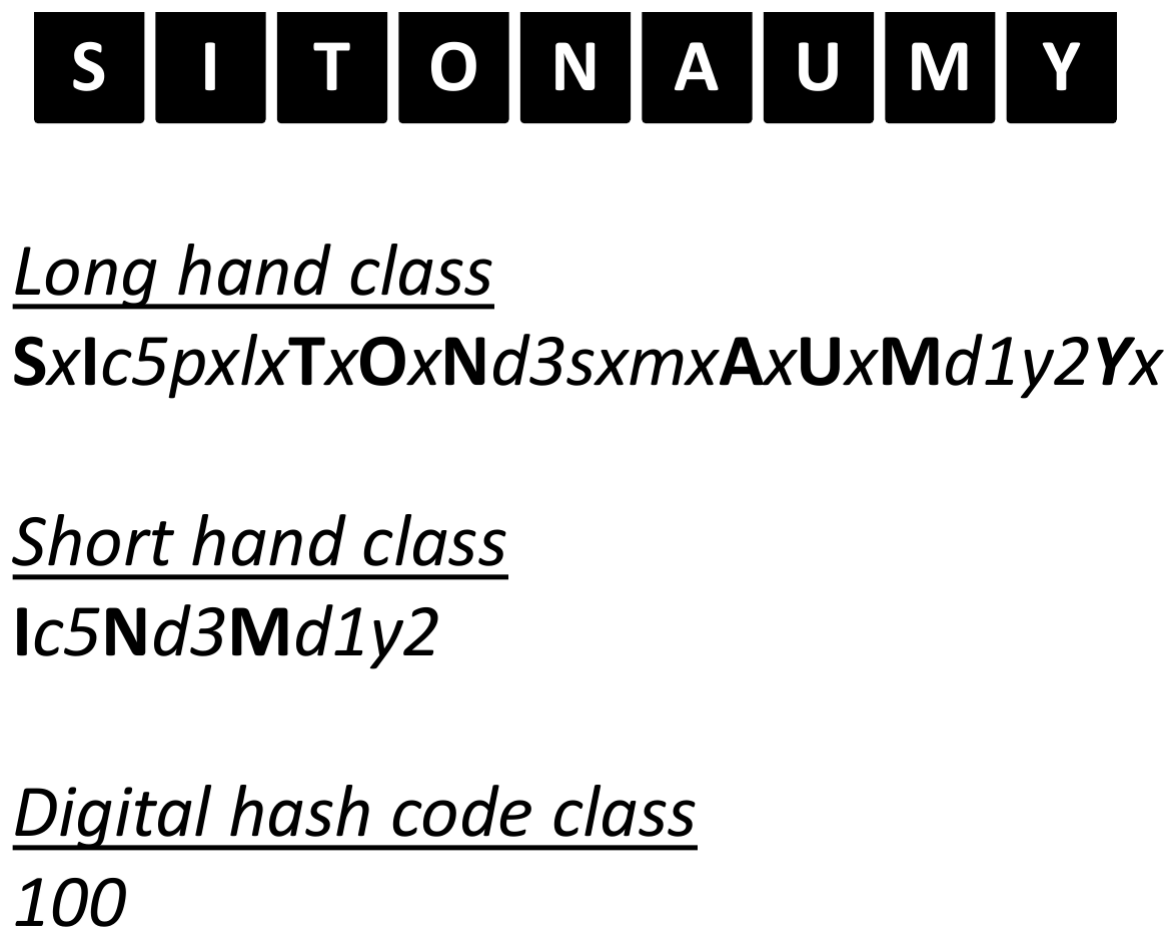
Taxonomy mnemonic and example of coding.

Classification of sedentary behaviors based on this structure could be achieved using a coding system based on a unique resource identifier (URI) similar to ISBN, as recommended by the Open Linked Data initiative (www.linkeddata.org). Each period of sedentary behavior could be labelled by a digital code that will describe it in detail and allow researchers to class behaviors in more or less refined categories, depending on need. To allow expansion and flexibility of the classification in the future, categories/facets/domains are not assigned a fixed number of digits. Instead the code is formed by simple catenation of the labels attached to the facets and categories (Figures 2-11) into a string (Figure 12). The example given (Figure 12) represents the coding of the most common type of sedentary behavior data available and codes a single bout of sedentary behavior, recorded with an actigraph accelerometer, in an urban population, on the morning of a summer day, with no other available information. Faceted complementary domains are represented by letters. The level one super-facets (purpo**S**e, env**I**ronment, pos**T**ure, s**O**cial, measureme**N**t, **A**ssociated behavior, stat**U**s, ti**M**e, t**Y**pe) form the upper-case mnemonic SITONAUMY. Complementary sub-categories are represented by lower case letters. Mutually exclusive categories are coded with integer numbers. The number zero universally represents an “other” category. The letter “x” universally signifies an undefined domain/categories (for which no information exists in the data). Domains followed by zero and “x” can be omitted to form a short-hand classification. It is proposed that this short-hand class is then automatically converted to a unique digital identifier (hash code), using standardised W3C conventions (www.w3c.org) for use in database and analysis software and to facilitate data sharing, aggregation and mining. 

### Remaining Issues

While a consensus structure and facets to classify sedentary behaviors has been achieved there are some remaining issues. The consensual classification system allows common cross-disciplinary terminology, flexibility as research advances and does not impose a set ontology - but it could potentially lead to a very large set of classes. Advances in the field of sedentary behavior research and the folksonomy will surely help to trim down the number of classes needed but schemes to limit the number of classes should be the focus of Round 2 of this consensual process. The system is also not entirely free of ambiguity. For example, how would “two friends eating a meal in front of the television in a fast food restaurant?” be classified. This is ambigous as “Eating” falls under the Purpose facet but also under the Associated Behavior facet and the social nature falls under the Social facet and Purpose facet. Refining the vocabulary might help in lifting some of these ambiguities and ensure consistency in classification (eg. using “meal” instead of “eating” for purpose and “snacking” instead of “eating” in associated behavior). Testing for and removing ambiguity should also be a focus of Round 2.

### Round 2

This draft taxonomy will be presented to the pool of experts contacted in Round 1 and those registered on the website, for review. In addition a new scan for authors of research on sedentary behavior will be conducted and the author contacted directly. Opinion about the proposed taxonomy and suggested alteration and refinement will be collected through an online survey and direct communications. It is hoped that other experts and opinions can be gathered through using the unique commenting feature of PLOS ONE. 

We would like to draw particular attention to the issues raised above and invite responses.

## Conclusion

There is general consensus that a taxonomy of sedentary behavior will help advances in the multi-disciplinary field of sedentary behavior research. There is also a consensus that such a taxonomy should be flexible to accommodate diverse purposes of use, and future advances in the field and yet act as a common language across all fields of research concerned with sedentary behavior. A consensus was reached on the taxonomy structure in the first Delphi Round enabling exhaustive inclusion and organisation of all proposed facets. It synthesises current views and opinions of a broad research community and diverse ontology. The structure appears to fit the criteria demanded by experts and to allow mapping the taxonomy on current broader frameworks and models of sedentary behavior and linkage to quantification frameworks for surveillance, such as SITT and the WHO ICF. 

## Supporting Information

Text S1 **Document detailing types of taxonomy architecture.**
(DOCX)

Figure S1 **Schematic examples of the four most common types of taxonomy structures.**
(TIF)
