# Correction: Development of a Consensus Taxonomy of Sedentary Behaviors (SIT): Report of Delphi Round 1

**DOI:** 10.1371/annotation/40e70c58-2067-4211-a152-22c3ab5534f5

**Published:** 2014-01-07

**Authors:** Sebastien Francois Martin Chastin, Ulf Schwarz, Dawn A Skelton

The name of the last author is incorrect. The correct name is: Dawn A Skelton.

