# Supplementary material for: Development of a Consensus Taxonomy of Sedentary Behaviors (SIT): Report of Delphi Round 1
Source: PLoS One. 2013 Dec 2;8(12):e82313. doi: 10.1371/journal.pone.0082313 (PMC3847079; doi:10.1371/journal.pone.0082313)
Supplement: Text S1 — Document detailing types of taxonomy architecture. (DOCX) [file pone.0082313.s001.docx]

**Supporting INFORMATION**

**Type of taxonomy architectures**

The architecture of any taxonomy strongly dictates how it can be used. So it is important that the architecture is fit for purpose and allows full achievement of the criteria set for the classification. Experts were presented with the four most common taxonomy structures (flat, hierarchical, faceted and networked) shown in figure S1. A concise definition and examples for each of these architectures are given below.

*Flat structure*

Flat taxonomies group content into a controlled set of categories which are mutually exclusive. There is no relationship among the categories. A typical example of flat taxonomy is the compendium of physical activity (<https://sites.google.com/site/compendiumofphysicalactivities>) which organises physical activities in 21 separate categories such as: cycling, conditioning exercise and home repair. In this structure, a sedentary behavior will have to be classed into a single category and this might create some conflicts. For example, how would an instance of sitting while driving for work purposes be classed in the flat structure of Figure S1?

*Hierarchical structure*

This is the most well known form of taxonomy used, for example, in the classification of biological species. It has a tree structure with nodes and vertices, with the vertices representing the relationship between a parent and child category. Moving up the hierarchy means expanding or broadening the category. Moving down the hierarchy means refining or qualifying the category. So developing a hierarchical taxonomy requires a definition of domain/sub-domain/categories/subcategories and explicit rules to explain their hierarchical dependencies. This means that the characteristics which define the parent levels of the hierarchy have, by default, more importance than those defining the child levels. To develop this type of structure it is, therefore, necessary to define an order of importance for the characteristics of sedentary behaviours. In the example of hierarchical structure in Figure S1, this means defining work-based sedentary behaviour as more important than computer use.

*Faceted structure*

In a faceted taxonomy multiple characteristics (facets) can be assigned to an object, instance, event etc (the content) that need to be classified. This set of facets defines the content. The Dewey or ISBN systems for classification of books are well known examples of faceted taxonomies. In these, books are classified using an author facet, a subject facet, a date facet, publisher facet etc. It is similar to a flat structure but content does not belong exclusively to a single category. Therefore the kind of classification conflicts that might arise in a flat structure do not exist in a faceted structure. Unlike hierarchical structures, a faceted structure does not require the definition of relationships or the need to apply greater importance to one characteristic over another. Faceted taxonomies are more flexible but can rapidly become complex.

*Networked taxonomy*

Similar to the hierarchical structure but each node can have multiple parents and children. The vertices between nodes represent relationships between domains which can be hierarchical dependencies or can reflect associations between domains. It is less intuitive and more difficult to represent graphically, but offers a lot more flexibility. In the networked taxonomy in Figure S1, the categories for driving and computer use are the ‘children’ of the seated posture category. However, they can be linked through the work relationship (dashed line). An example of networked taxonomy is the gene ontology (GO).

**Figure legends**

Figure S1: Schematic examples of the four most common types of taxonomy structures (flat, hierarchical, faceted and networked) in the context of sedentary behavior.
