# Supplementary figures and images for: Development of a Consensus Taxonomy of Sedentary Behaviors (SIT): Report of Delphi Round 1
Source: PLoS One. 2013 Dec 2;8(12):e82313. doi: 10.1371/journal.pone.0082313 (PMC3847079; doi:10.1371/journal.pone.0082313)

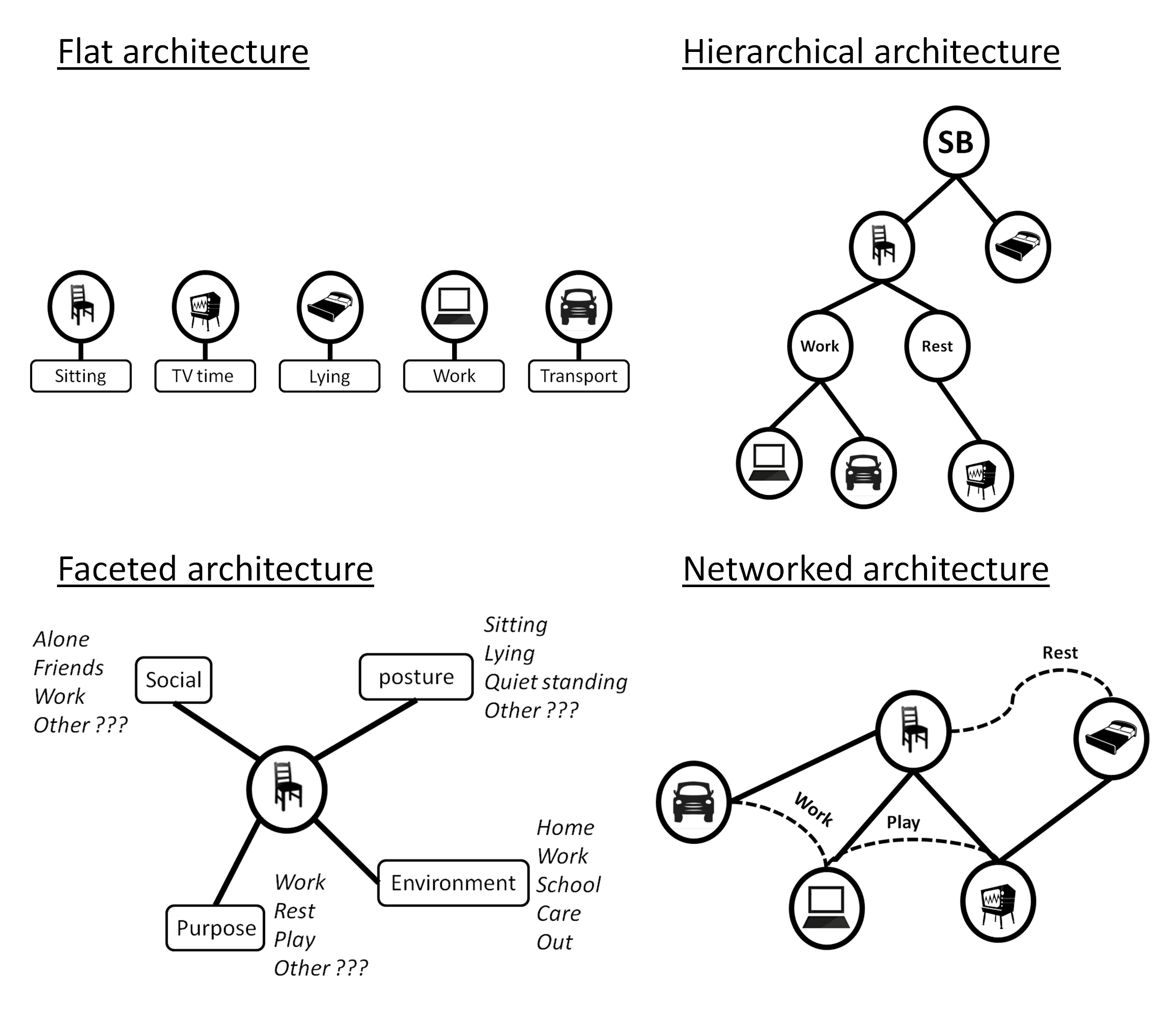

Supplement: Figure S1 — Schematic examples of the four most common types of taxonomy structures. (TIF) [file pone.0082313.s002.tif]
